# Treatment of Patients With Late-Stage Colorectal Cancer: ASCO Resource-Stratified Guideline

**DOI:** 10.1200/JGO.19.00367

**Published:** 2020-03-09

**Authors:** E. Gabriela Chiorean, Govind Nandakumar, Temidayo Fadelu, Sarah Temin, Ashley Efrain Alarcon-Rozas, Suyapa Bejarano, Adina-Emilia Croitoru, Surbhi Grover, Pritesh V. Lohar, Andrew Odhiambo, Se Hoon Park, Erika Ruiz Garcia, Catherine Teh, Azmina Rose, Bassem Zaki, Mary D. Chamberlin

**Affiliations:** ^1^ University of Washington, Fred Hutchinson Cancer Research Center, Seattle, WA; ^2^ Columbia Asia Hospitals, Bangalore, India; ^3^ Weill Cornell Medical College, New York, NY; ^4^ Dana-Farber Cancer Institute, Boston, MA; ^5^ American Society of Clinical Oncology, Alexandria, VA; ^6^ Clinica Angloamericana, Lima, Peru; ^7^ Excelmedica, Liga Contra el Cancer Honduras, San Pedro Sulal, Honduras; ^8^ Fundeni Clinical Institute, Bucharest, Romania; ^9^ University of Pennsylvania, Philadelphia, PA; ^10^ HCG Cancer Center, Vadodara, Gujarat, India; ^11^ University of Nairobi, College of Health Sciences, Nairobi, Kenya; ^12^ Samsung Medical Center, Seoul, South Korea; ^13^ Insituto Nacional De Cancerologia, Mexico City, Mexico; ^14^ Philippine Association of HPB Surgeons/Makati Medical Center, Makati City, Philippines; ^15^ Independent Colorectal Patient Representative, London, United Kingdom; ^16^ Dartmouth-Hitchcock Medical Center, Lebanon, NH

## Abstract

**PURPOSE:**

To provide expert guidance to clinicians and policymakers in resource-constrained settings on the management of patients with late-stage colorectal cancer.

**METHODS:**

ASCO convened a multidisciplinary, multinational Expert Panel that reviewed existing guidelines, conducted a modified ADAPTE process, and used a formal consensus process with additional experts for two rounds of formal ratings.

**RESULTS:**

Existing sets of guidelines from four guideline developers were identified and reviewed; adapted recommendations from five guidelines form the evidence base and provided evidence to inform the formal consensus process, which resulted in agreement of ≥ 75% on all recommendations.

**RECOMMENDATIONS:**

Common elements of symptom management include addressing clinically acute situations. Diagnosis should involve the primary tumor and, in some cases, endoscopy, and staging should involve digital rectal exam and/or imaging, depending on resources available. Most patients receive treatment with chemotherapy, where chemotherapy is available. If, after a period of chemotherapy, patients become candidates for surgical resection with curative intent of both primary tumor and liver or lung metastatic lesions on the basis of evaluation in multidisciplinary tumor boards, the guidelines recommend patients undergo surgery in centers of expertise if possible. On-treatment surveillance includes a combination of taking medical history, performing physical examinations, blood work, and imaging; specifics, including frequency, depend on resource-based setting.

Additional information is available at www.asco.org/resource-stratified-guidelines.

## INTRODUCTION

The purpose of this guideline is to provide expert guidance on the treatment and follow-up of patients with late-stage colorectal cancer to clinicians, public health leaders, and policymakers in all resource settings. The target population is people with late-stage colorectal cancer (metastatic TNM stage: T any, N any, M1; or unresectable TNM stage: T any, N any, M0 colon cancer or rectal cancer).

Historically, some of the highest incidence rates have been in regions described as more developed, including North America, Australia/New Zealand, Europe, Japan, and South Korea. However, in 2012, approximately 45% of incident colorectal cancers occurred in less-developed regions (the term often overlaps with the term low- and middle-income countries [LMICs]) around the world, representing 9%-10% of cancers in those regions.^1^ Fifty-two percent of deaths from colorectal cancer occurred in these less-developed regions. In 2018, GLOBOCAN presented its data in terms of the Human Development Index (HDI), rather than by income, and showed that the highest incidence and mortality was in high/very high HDI regions (Table 1). In some more developed regions, rates are decreasing.^2^

**TABLE 1:**
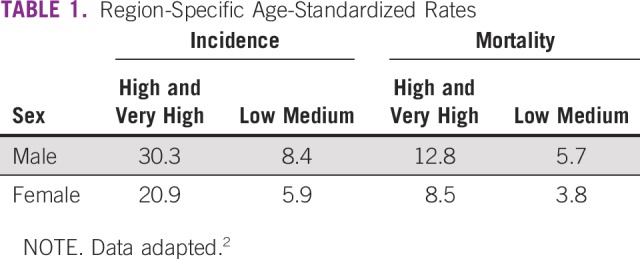
Region-Specific Age-Standardized Rates

Some of these numbers are increasing in some parts of the world (eg, increases in cases and deaths in some Eastern European countries, in some South American countries, and China). Rates are lowest in most regions of Africa and Southern Asia.^2^ Different regions of the world, both among and within countries, differ with respect to access to early detection. Many regions do not have mass or even opportunistic screening, and even within regions with mass screening, subpopulations may not have access to screening. As a result of these disparities, the ASCO Resource-Stratified Guidelines Advisory Group chose colorectal cancer as a priority topic for guideline development.

**THE BOTTOM LINE**

**Treatment of Patients with Late-Stage Colorectal Cancer: ASCO Resource-Stratified Guideline**

**Guideline Question**

For each of the resource settings, what is the optimal treatment of patients with late-stage colorectal cancer from initial diagnosis to follow-up?

**Target Population**

Patients with late-stage colon cancer and patients with late-stage rectal cancer.

**Target Audience**

Experts in medical oncology, radiation oncology, surgery, surgical oncology, gastroenterology, statistics, and nonmedical community members, including patients and member(s) of advocacy groups.

**Methods**

A multinational, multidisciplinary Expert Panel was convened to develop clinical practice guideline recommendations based on a systematic review of existing guidelines and a formal consensus process.

**Key Recommendations**

***Clinical Question 1***

What are the optimal methods of initial symptom management, diagnosis, and staging for patients with late-stage colorectal cancer?

- In basic and limited settings, symptom management includes: symptom control, surgical evaluation, transfusion, palliative care.
- Diagnosis includes biopsy, pathology, endoscopy (in limited settings only). Options discussed include endoscopy, digital rectal exam (DRE), and imaging, dependent on resource settings.

See Tables 3-5 for full list of recommendations.

***Clinical Question 2***

What are the optimal systemic treatments for patients with late-stage colorectal cancer in first line?

- Most patients receive treatment with chemotherapy, where chemotherapy is available.
- If, after a period of chemotherapy, patients become candidates for surgical resection with curative intent of both primary tumor and liver or lung metastatic lesions based on evaluation in multidisciplinary tumor boards, patients are recommended to undergo surgery in centers of expertise.

See Table 6 for full list of recommendations.

***Clinical Question 3***

What are the optimal treatments for patients with late-stage colorectal cancer who have received one prior line of therapy?

- In enhanced and maximal settings, chemotherapy is recommended and is conditional upon what patients received in the first line.

See Table 7 for full list of recommendations.

***Clinical Question 4***

What are the optimal treatments for patients with late-stage colorectal cancer who have received two prior lines of therapy?

- In maximal settings, systemic therapy options are presented and are conditional upon prior treatment.

See Table 8 for full list of recommendations.

***Clinical Question 5***

What are selected liver-directed therapy options for patients with late-stage colorectal cancer and liver metastases?

- In maximal settings only, for patients with liver metastases, options are presented. Recommendations should be implemented in centers of expertise in the specific technique after multidisciplinary review, or in the context of a clinical trial.

See Table 9 for full list of recommendations.

***Clinical Question 6***

What is a summary of the optimal treatments for patients with late-stage colorectal cancer?

- In basic and limited settings, if high risk of obstruction, significant bleeding, perforation, or tumor-related symptoms: resection of primary tumor OR if obstruction from primary tumor or from peritoneal metastases: diverting ostomy.
- In enhanced and maximal settings, the guideline adds option of colon or rectal stenting.
- In enhanced and maximal settings only, if patients with metastatic rectal cancer have a symptomatic primary rectal tumor, radiation therapy (± chemotherapy) should be discussed.
- Patients who have received surgery/ablation may receive systemic therapy if available (enhanced/maximal).

See Table 10 for full list of recommendations by modality

***Clinical Question 7***

What are the optimal on-treatment surveillance and follow-up strategies for patients treated for mCRC?

- On-treatment surveillance includes a combination of taking the medical history, performing physical examinations, blood work, and imaging; specifics, including frequency, depend on resource-based setting.

See Table 11 for full list of recommendations.

**Additional Resources**

More information, including a data supplement with additional evidence tables, slide sets, and clinical tools and resources, is available at www.asco.org/resource-stratified-guidelines. The Methodology Manual (available at www.asco.org/guideline-methodology) provides additional information about the methods used to develop this guideline. Patient information is available at www.cancer.net.

**All recommendations underwent Formal Consensus.**

**ASCO believes that cancer clinical trials are vital to inform medical decisions and improve cancer care, and that all patients should have the opportunity to participate.**

ASCO has established a process for development of resource-stratified guidelines, which includes mixed methods of evidence-based guideline development, adaptation of the clinical practice guidelines of other organizations, and formal expert consensus. This article summarizes the results of that process and presents resource-stratified recommendations, which are based in part on formal expert consensus and adaptation from existing guidelines (see Results section and Appendix Table A1).

In developing resource-stratified guidelines, ASCO has adopted its framework from the four-tier resource setting approach (basic, limited, enhanced, maximal; Table 2) developed by Breast Health Global Initiative and modifications to that framework based on the Disease Control Priorities 3.^3,4^ The framework emphasizes that variations occur not only between but within countries with disparities, for example, between rural and urban areas.

**TABLE 2:**
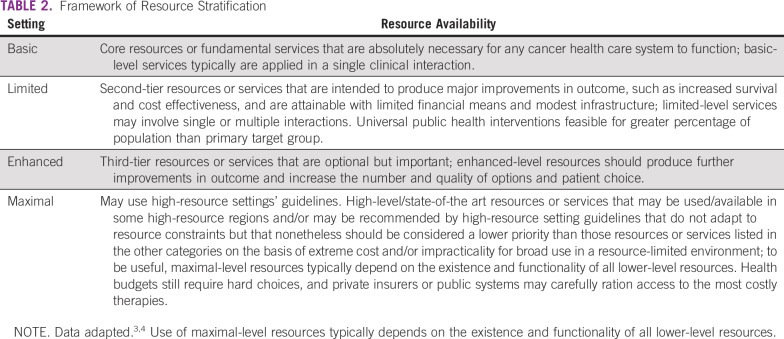
Framework of Resource Stratification

## GUIDELINE QUESTIONS

This clinical practice guideline addresses seven clinical questions in each resource setting: (1) What are the optimal methods of initial symptom management, diagnosis, and staging? (2) What are the optimal systemic treatments for patients with late-stage colorectal cancer in first line? (3) What are the optimal treatments for patients with late-stage colorectal cancer who have received one prior line of therapy? (4) What are the optimal treatments for patients with late-stage colorectal cancer who have received two prior lines of therapy? (5) What are selected liver-directed therapy options for patients with late-stage colorectal cancer and liver metastases? (6) What is a summary of the optimal treatments for patients with late-stage colorectal cancer? (7) What are the optimal on-treatment surveillance and follow-up strategies for patients treated for metastatic colorectal cancer (mCRC)?

## METHODS

### Guideline Development Process

This systematic review–based guideline was developed by a multidisciplinary Expert Panel, which included a patient representative and an ASCO guidelines staff with health research methodology expertise (Appendix Table A2). The Expert Panel met once in person and otherwise via teleconference and/or webinar and corresponded through e-mail. Based upon the consideration of the evidence, the authors were asked to contribute to the development of the guideline, provide critical review, and finalize the guideline recommendations. This guideline was partially informed by ASCO’s modified-Delphi Formal Expert Consensus methodology, during which the Expert Panel was supplemented by additional experts recruited to rate their agreement with the drafted recommendations. The Expert Panel and the additional experts are referred to as the Consensus Panel (Data Supplement). The guideline recommendations were sent for an open comment period of 2 weeks, allowing the public to review and comment on the recommendations after submitting a confidentiality agreement. These comments were taken into consideration while finalizing the recommendations. Members of the Expert Panel were responsible for reviewing and approving the penultimate version of guideline, which was then circulated for external review and submitted to an ASCO journal for editorial review and consideration for publication. All ASCO guidelines are ultimately reviewed and approved by the Expert Panel and the ASCO Clinical Practice Guidelines Committee prior to publication. All funding for the administration of the project was provided by ASCO.

ASCO’s adaptation and formal consensus processes begin with a literature search to identify candidate guidelines for adaptation. The review of candidate guidelines includes two parts: methodological review and content review.^5^ The methodological review was completed by ASCO senior guideline staff. The content review was completed by the ASCO Expert Panel.

The guideline recommendations were crafted, in part, using the Guidelines Into Decision Support (GLIDES) methodology and accompanying BRIDGE-Wiz software.^6^ Detailed information about the methods used to develop this guideline is available in the ASCO Guidelines Methodology Manual (available at www.asco.org/guideline-methodology) and the Data Supplement at www.asco.org/resource-stratified-guidelines.

The ASCO Expert Panel and guidelines staff will work with co-chairs to keep abreast of any substantive updates to the guideline. Based on formal review of the emerging literature, ASCO will determine the need to update. The ASCO Guidelines Methodology Manual provides additional information about the guideline update process. This is the most recent information as of the publication date.

### Guideline Disclaimer

The Clinical Practice Guidelines and other guidance published herein are provided by the American Society of Clinical Oncology, Inc. (ASCO) to assist providers in clinical decision making. The information herein should not be relied upon as being complete or accurate, nor should it be considered as inclusive of all proper treatments or methods of care or as a statement of the standard of care. With the rapid development of scientific knowledge, new evidence may emerge between the time information is developed and when it is published or read. The information is not continually updated and may not reflect the most recent evidence. The information addresses only the topics specifically identified therein and is not applicable to other interventions, diseases, or stages of diseases. This information does not mandate any particular course of medical care. Further, the information is not intended to substitute for the independent professional judgment of the treating provider, as the information does not account for individual variation among patients. Recommendations reflect high, moderate, or low confidence that the recommendation reflects the net effect of a given course of action. The use of words like “must,” “must not,” “should,” and “should not” indicates that a course of action is recommended or not recommended for either most or many patients, but there is latitude for the treating physician to select other courses of action in individual cases. In all cases, the selected course of action should be considered by the treating provider in the context of treating the individual patient. Use of the information is voluntary. ASCO provides this information on an “as is” basis and makes no warranty, express or implied, regarding the information. ASCO specifically disclaims any warranties of merchantability or fitness for a particular use or purpose. ASCO assumes no responsibility for any injury or damage to persons or property arising out of or related to any use of this information, or for any errors or omissions.

### Guideline and Conflicts of Interest

The Expert Panel was assembled in accordance with ASCO’s Conflict of Interest Policy Implementation for Clinical Practice Guidelines (“Policy,” found at http://www.asco.org/rwc). All members of the Expert Panel completed ASCO’s disclosure form, which requires disclosure of financial and other interests, including relationships with commercial entities that are reasonably likely to experience direct regulatory or commercial impact as a result of promulgation of the guideline. Categories for disclosure include employment; leadership; stock or other ownership; honoraria, consulting or advisory role; speaker’s bureau; research funding; patents, royalties, other intellectual property; expert testimony; travel, accommodations, expenses; and other relationships. In accordance with the Policy, the majority of the members of the Expert Panel did not disclose any relationships constituting a conflict under the Policy.

## RESULTS

### Literature Search

The recommendations were developed by using a systematic review of high-quality published guidelines and clinical experience. The Expert Panel conducted a search of systematic review–based guidelines published between 2012 and July 31, 2018 in PubMed, Standards and Guidelines Evidence directory,^7^ Cochrane Systematic Reviews and (the formerly extant) US National Guideline Clearinghouse (NGC) databases. Initial searches used for two previous ASCO Colorectal Cancer–related resource-stratified guidelines^8,9^ were used, updated, and complemented by searches of G-I-N International Guideline Library. Articles were selected for inclusion in the systematic review based on the following criteria: (1) addressed the diagnosis or treatment of late-stage colon and/or late-stage rectal cancer, (2) developed by multidisciplinary content experts as part of a recognized organizational effort, and (3) published between 2012 and 2018.

Articles were excluded from the systematic review if they were (1) meeting abstracts; or (2) books, editorials, commentaries, letters, news articles, case reports, or narrative reviews. After initial searches of primary literature, the panel leadership decided to primarily use guidelines to inform expert consensus. ASCO considered quality guidelines that either met the US National Guidelines Clearinghouse 2013 criteria as assessed by NGC or met ASCO criteria for Appraisal of Guidelines for Research and Evaluation II (AGREE II) methodologic review. Searches for cost-effectiveness analyses were also conducted separately.

A total of 10 guidelines were found in the literature searches from the following developers: National Comprehensive Cancer Network (NCCN) 2018,^10^ European Society for Medical Oncology (ESMO; two guidelines—rectal cancer^11^ and metastatic colorectal cancer^12^), Singapore Cancer Network (SCAN),^13^ Japanese Society for Cancer of the Colon and Rectum Guidelines (JSCCR),^14^ Spanish Society of Medical Oncology (SEOM),^15^ American Society of Colon and Rectal Surgeons (ASCR),^16,17^ Cancer Council Australia (CCA),^18^ a Pan-Asian group,^19^ and National Institute for Heath and Care Excellence (UK) (NICE).^20^ The ASCO Expert Panel reviewed seven of these guidelines in depth for their currency, content, and methodology (Cancer Council Australia, NCCN 2018, ESMO rectal, ESMO colon, JSCCR, SCAN, and NICE). On the basis of content and methodology reviews (the latter by either ASCO or the NGC), the Expert Panel chose five evidence-based guidelines from four public health authorities/guideline developers (Cancer Council Australia,^18^ ESMO,^11,12^ NCCN 2018,^10^ NICE^20^) as most relevant. Appendix Table A1 lists links to the guidelines. The Expert Panel used these guidelines, some literature suggested by the Expert Panel (eg, a consensus document from the Expert Group on OncoSurgery management of Liver Metastases; EGOSLIM),^21^ and clinical experience as guides. The Expert Panel formally vetted the included guidelines’ content and development methodology. The Expert Panel was aware of a 2019 NCCN guideline update available; however, it was not formally reviewed, as it was published after the prespecified closing date parameter of the literature search. The Data Supplement includes a detailed overview of the included guidelines, including information on the clinical questions, target populations, development methodology, and key evidence.

This ASCO guideline reinforces selected recommendations offered in the Cancer Council Australia, ESMO, NICE, and NCCN 2018 guidelines and acknowledges the effort put forth by the authors and aforementioned societies to produce evidence-based and/or consensus-based guidelines informing practitioners and institutions that provide care to patients with metastatic/late-stage colorectal cancer.

## GUIDELINES ON TREATMENT OF PATIENTS WITH METASTATIC COLORECTAL CANCER

The Expert Panel identified clinical questions and/or categories within the adapted guidelines that would potentially match the ASCO clinical questions. Most of the maximal setting guidelines did not explicitly label clinical questions, with the exception of the guideline by Cancer Council Australia. The target populations were all in maximal settings and included patients with advanced colorectal and/or specifically rectal cancer; some of the guidelines included sections on patients with liver metastases. Specific clinical questions (if provided) and target populations of the adapted guidelines are listed in the Data Supplement.

At the time of the systematic searches for quality existing guidelines for this ASCO Resource-Stratified Guideline, there was a paucity of systematic review–based clinical practice guidelines published in the date parameter (Data Supplement). Only two sections (of eight sections used) of the guidelines found were systematic review based (in the Cancer Council Australia guidelines). Other guidelines used other methods, such as nonsystematic literature searches and consensus. The key evidence the guidelines used included systematic reviews, meta-analyses, nonsystematic literature reviews, existing guidelines, and consensus. Most of the evidence regarded systemic therapy. In some areas regarding other interventions, the guidelines used observational data. For example, regarding curative surgery in patients with synchronous or metachronous metastases, Cancer Council Australia conducted a systematic review and found only cohort studies. Therefore, many recommendations in this ASCO guideline were informed by this variety of expert-reviewed data and then validated by Formal Consensus.

The outcomes/end points in most studies reviewed by the adapted guidelines included efficacy (including overall survival [OS] and disease-free survival), quality of life, safety/adverse events, and, in some cases, cost effectiveness.

## RESULTS OF ASCO METHODOLOGICAL REVIEW

The methodologic review of the guidelines was completed by two ASCO guideline staff members for each guideline using the Rigor of Development subscale of the AGREE II instrument (except those already assessed by NGC). The score for the Rigor of Development domain is calculated by summing the scores across individual items in the domain and standardizing the total score as a proportion of the maximum possible score. Detailed results of the scoring and the AGREE II assessment process for this guideline are available in the Data Supplement.

## RECOMMENDATIONS

The recommendations were developed by a multinational, multidisciplinary group of experts using evidence from existing guidelines and clinical experience as a guide. The ASCO Expert Panel underscores that health care practitioners who implement the recommendations presented in this guideline should first identify the available resources in their local and referral facilities and endeavor to provide the highest level of care possible with those resources.

### CLINICAL QUESTION 1

What are the optimal methods of initial **symptom management, diagnosis, and staging** for patients with late-stage colorectal cancer?

Recommendations on symptom management, diagnosis, and staging are provided in Tables 3, 4, and 5. These recommendations were adapted and, in some cases, modified from the guidelines from the following developers: NICE, ESMO, NCCN, and Cancer Council Australia, and informed by the EGOSLIM document.^21^ These recommendations also refer to two ASCO palliative care guidelines and an American Society for Clinical Pathology/College of American Pathologists/Association for Molecular Pathology/ASCO molecular biomarker evaluation guideline.^22-24^

**TABLE 3:**
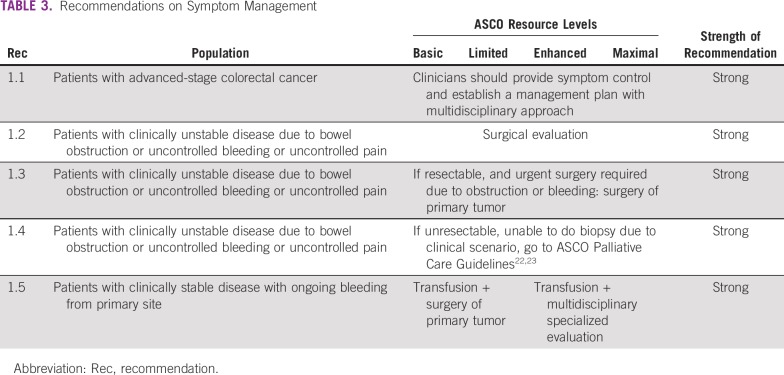
Recommendations on Symptom Management

**TABLE 4:**
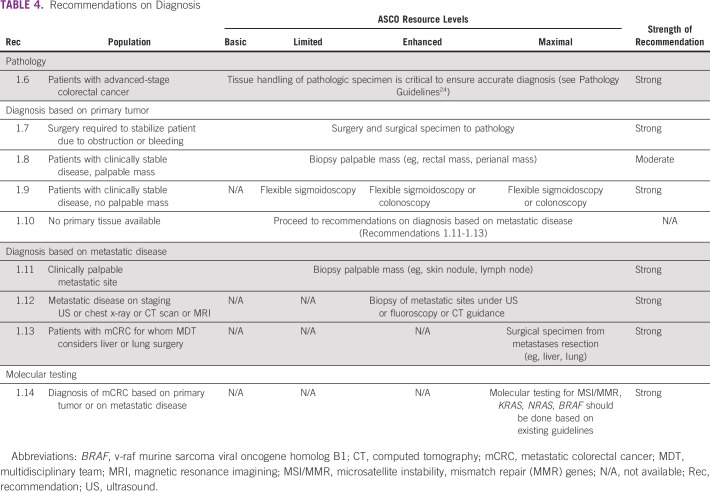
Recommendations on Diagnosis

**TABLE 5:**
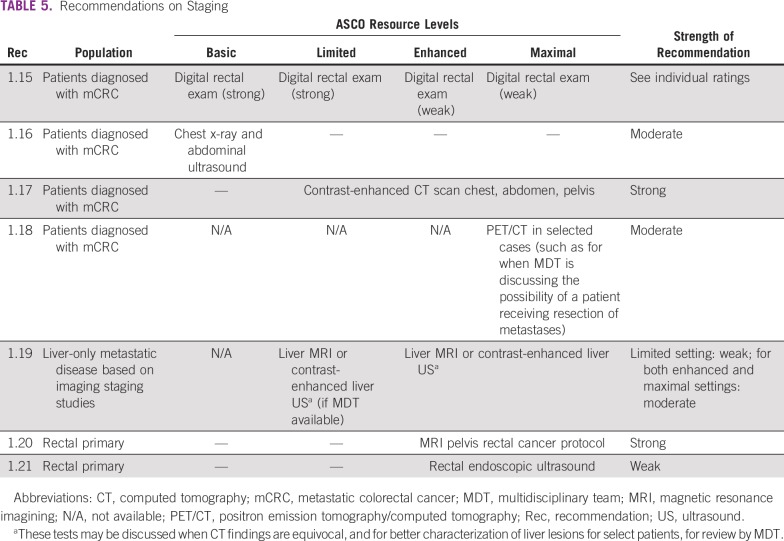
Recommendations on Staging

### Symptom Management

Recommendations for assisting patients with symptoms of advanced colorectal cancer such as pain or bleeding are in Table 3.

#### Discussion.

More than 1.8 million patients in the world were diagnosed with colorectal cancer (CRC) in 2018. Among all patients with CRC, 20%-30% have metastatic disease from the outset (synchronous primary tumor and metastatic disease). Of those patients who present with local disease, 50%-60% will develop metastatic disease (metachronous metastases) within the first 3 years of diagnosis.^1,25-27^

The first priority for clinicians with any patient with advanced cancer is to provide symptom management according to ASCO Palliative Care Guidelines.^22,23^

If a patient is clinically unstable, for example with bowel obstruction, impending perforation, uncontrolled bleeding, and/or uncontrolled pain, surgeons may need to perform emergency colon or rectal cancer surgery. If resection is not possible, then patients should receive palliative care.^12,18^ Palliative colostomy should be considered in situations of malignant bowel obstruction. In the assessment of general symptoms, clinicians should determine a patient’s performance status and comorbid conditions, as they can influence the ability to receive and predict the benefit from medical treatment.

### Diagnosis

Recommendations on the methods of diagnosis for patients with colorectal cancer are in Table 4.

#### Discussion.

The purpose of the initial workup of patients with suspected mCRC is to confirm the pathologic diagnosis by histology, immunohistochemistry, and/or molecular markers as indicated (Table 4). These elements guide treatment options (via predictive markers) and have prognostic value. A comprehensive clinical evaluation of symptoms, general health status, and comorbid conditions are also key components of the workup for patients with late-stage CRC.

Obtaining a pathologic diagnosis is essential; however, the Expert Panel recognizes there may be limited or no access to imaging (to perform guided biopsies) and pathology services in some resource-constrained settings. Histologic diagnosis is performed on tissue samples obtained through a biopsy from the primary tumor or metastatic site, depending on accessibility of tumor and resource availability. In basic settings, digital rectal exam and barium enema may be helpful in identifying a primary tumor in the rectum or sigmoid colon and to determine risk of obstruction. Palpable primary tumors (eg, distal rectal, anal canal), or metastatic sites (eg, lymph nodes, skin nodules) may be amenable to direct local biopsy in basic settings. In limited settings, flexible sigmoidoscopy can be used to identify and biopsy primary tumors located in the descending colon, sigmoid colon, and rectum. In enhanced and maximal settings, colonoscopy is available to identify and biopsy tumors throughout the colon and rectum. Biopsy of metastatic sites under ultrasound or computed tomography (CT) guidance is only available in enhanced and maximal settings.^8^

Where pathology services are established, treating clinicians and pathologists should ensure proper tissue handling and accurate tissue examination for accurate diagnosis. Pathology is outside the scope of this guideline, but laboratories should follow quality pathology standards outlined by existing guidance documents.

When possible, all patients with mCRC should be tested for key molecular markers, if treatments are available based on such molecular marker results, before clinicians obtain these tests. Molecular markers that may be evaluated in maximal settings including, for example, mismatch repair protein (MMR)/microsatellite instability (MSI) status (microsatellite stable [MSS] *v* microsatellite instability high [MSI-high]), RAS, and BRAF (v-raf murine sarcoma viral oncogene homolog B1). Novel molecular markers are continuously being evaluated in advanced colorectal cancer, and they should only be tested in maximal settings, where targeted treatments are available, or clinical trials, as they can inform treatment options.^10,12,18^ It is not within the scope of this guideline to review the evidence on molecular markers, and the Expert Panel refers readers to separate systematic review–based guidelines on markers with the highest clinical utility as well as to best pathology practices.^24^ These markers may be helpful in choosing the best options for first-, second-, or third-line treatments but are only available in maximal and some enhanced-resource settings.

### Staging

Recommendations for staging are in Table 5.

#### Discussion.

Adequate staging of suspected late-stage CRC depends on imaging resources available. ESMO recommends a “stepwise imaging approach.”^12 (p1,397)^ In basic settings, chest x-ray and abdominal ultrasound are typically the only imaging modalities available. Limited settings may have contrast-enhanced CT scans of chest, abdomen, and pelvis available. Liver magnetic resonance imaging (MRI) or contrast-enhanced liver ultrasound for better evaluation of potentially resectable liver metastases should be available in enhanced- and maximal-resource settings where metastatic resections are being discussed. Liver MRI may assist when there are equivocal CT findings.

For best evaluation of rectal primary tumors, pelvis MRI rectal protocol (preferred), or rectal endoscopic ultrasound may be available in enhanced and maximal settings. Positron emission tomography (PET)/CT scans may also be available in maximal-resource settings for evaluation of metastatic disease, especially for patients deemed potential candidates for curative-intent surgery.^8^

Although it is critical to improving patients’ survival to identify resectable metastases, most patients with mCRC (90%) have unresectable disease at presentation. Chemotherapy may be very effective to convert the unresectable disease to potentially resectable disease. Therefore, for determining suitability for resection and the ultimate selection for surgery of patients with mCRC and for those with locally advanced, recurrent, or initially deemed inoperable CRC, assessment by multidisciplinary teams (MDTs) is critical.

Resectability of the primary tumor and of limited areas of metastatic disease in the liver or lung is determined by an MDT including specialized surgical consultation. In basic-resource settings, due to lack of accurate imaging, it is not possible for clinicians to determine if a patient’s late-stage CRC is resectable versus nonresectable. For determination of whether a patient is eligible for so-called curative-intent treatment, evaluation of the location and extent of primary tumor and distant metastases should be done where imaging is available either locally or by referral to centers of expertise. It is critical for an MDT to evaluate a patient, to determine eligibility for curative-intent treatment of all sites of disease and the best treatment sequence (eg, surgery upfront of primary tumor and metastatic sites followed by adjuvant chemotherapy, or neoadjuvant chemotherapy [NACT] followed by surgery of primary tumor and liver or lung metastatic sites, or perioperative chemotherapy before and after surgery). If practices or medical systems do not have MDTs, they should endeavor to develop them or refer the patient to a center with enhanced or maximal resources and MDT(s).^28^

Concurrent pain and symptom management are indicated whether a patient is receiving palliative care, chemotherapy, liver-directed therapy, or other curative-intent interventions.^23^ The most important factors driving clinicians’ recommendations for treatment of late-stage CRC, independent of resources, are, at a minimum: the patients’ clinical performance status (Eastern Cooperative Oncology Group Performance Status), general health/comorbidities, and pathology results. In enhanced and maximal-resource settings, the extent and resectability of distant metastases, right versus left side of the primary tumor, and the status of key molecular biomarkers are critical for assuring the best treatment and outcomes.

### Treatment

#### Discussion.

Most patients with late-stage (locally advanced inoperable or metastatic) CRC benefit from treatment with systemic chemotherapy. The choice of chemotherapy depends on resources available, toxicity concerns, whether the patient already had prior chemotherapy, and goals of care. Although there are several chemotherapy regimen options, including single agents or combination therapies, the best outcomes are achieved when patients can be exposed sequentially to the most combinations of available chemotherapy agents during their disease course (Tables 6-8).^29^

**TABLE 6:**
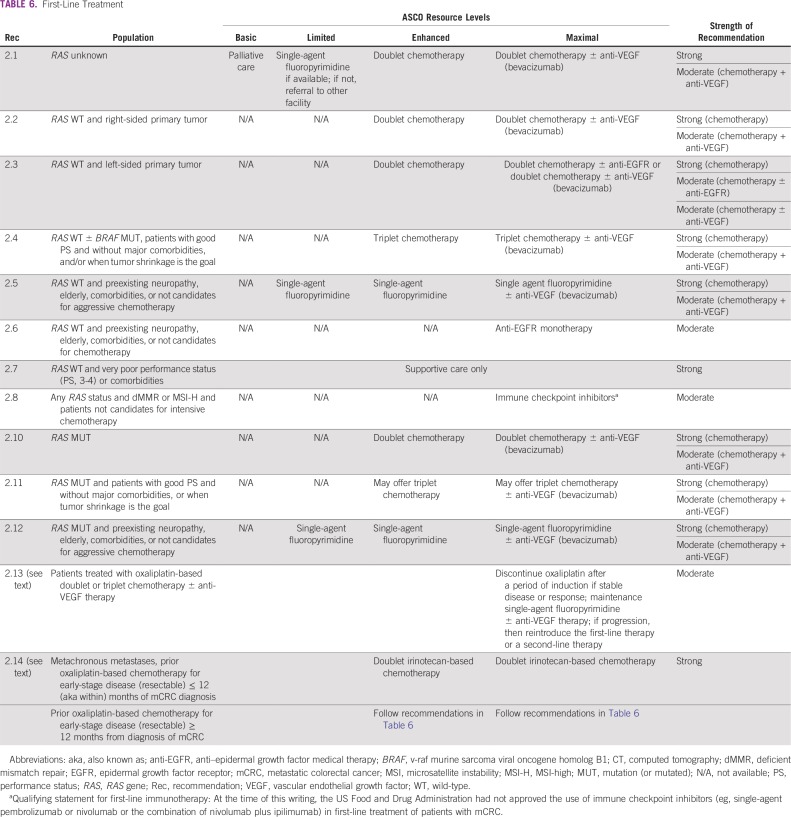
First-Line Treatment

**TABLE 7:**
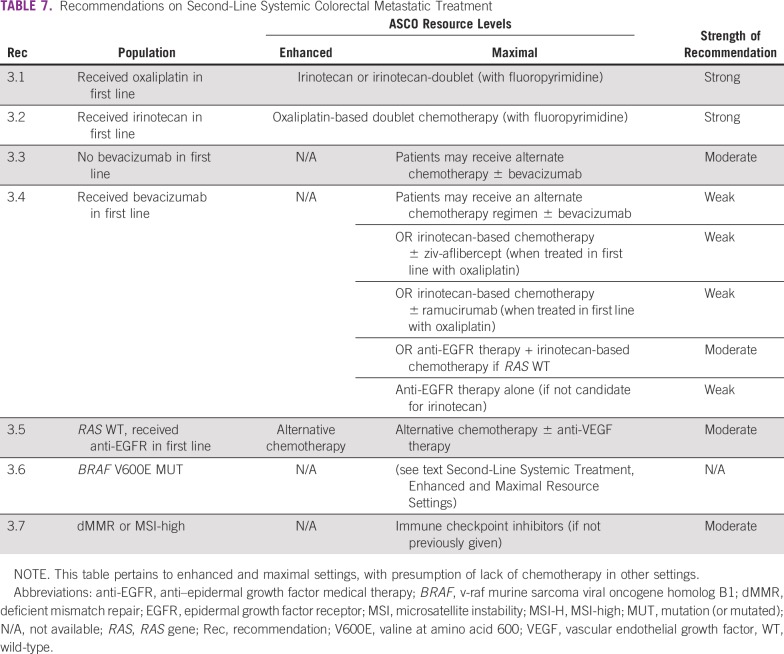
Recommendations on Second-Line Systemic Colorectal Metastatic Treatment

**TABLE 8:**
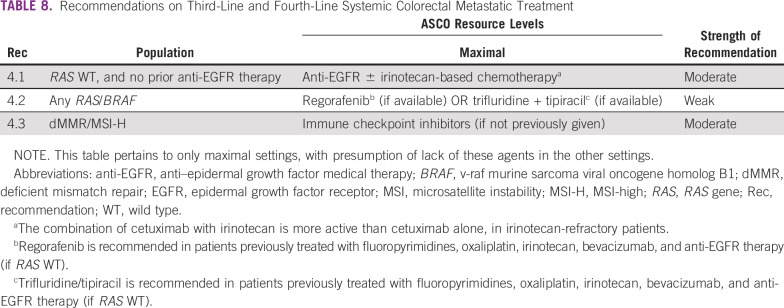
Recommendations on Third-Line and Fourth-Line Systemic Colorectal Metastatic Treatment

Practitioners administering the chemotherapy must know the intent of chemotherapy at all times. If the goal is to convert a patient’s unresectable disease to resectable disease, close follow-up is required, with clinical and radiographic assessments every 1-2 months.^12^ If the goal is palliation, then close clinical follow-up to determine clinical benefit is critical to avoid overtreatment and toxicity. Careful training of staff on safe handling and administration of chemotherapy and other cancer treatments is a key component of a successful cancer control program. Specialized cancer care can be delivered through a structure of task shifting, institutional twinning, and capacity building when oncologists are not available or in short supply.^30^

The toxic effects of antineoplastic drugs used for cancer treatment have been well known since their introduction in the 1940s. Therefore, patient safety during chemotherapy administration is essential and requires careful training and safety infrastructure for safe handling. Beyond the patient safety concerns, the occupational risks to health care workers handling these drugs in the course of their duties also need to be fully addressed.^31,32^ The Pan American Health Organization and WHO have made a publication available online entitled Safe Handling of Hazardous Chemotherapy Drugs in Limited-Resource Settings.^33^ ASCO has also published relevant handling standards (https://www.asco.org/practice-guidelines/quality-guidelines/standards): Chemotherapy Administration Safety Standards, including Standards for Pediatric Oncology^31^ and Safe Handling of Hazardous Drugs: ASCO Standards.^32^

Basic settings do not typically have any chemotherapy available. Limited-resource settings should have chemotherapy with fluoropyrimidines: 5-fluorouracil (FU) and/or capecitabine (Table 6). Enhanced-resource settings should have chemotherapy with FU, capecitabine, oxaliplatin, and irinotecan and should be able to treat patients with doublet (leucovorin calcium, FU, oxaliplatin [FOLFOX], capecitane, oxaliplatin [CAPEOX], leucovorin calcium, FU, irinotecan hydrochloride [FOLFIRI]) and/or triplet (leucovorin calcium, FU, oxaliplatin, irinotecan hydrochloride [FOLFOXIRI]) chemotherapy regimens when appropriate. The other types of systemic therapy include anti–vascular endothelial growth factor/receptor (VEGF/VEGFR) agents (eg, bevacizumab-preferred, ziv-aflibercept, ramucirumab, regorafenib), anti–epidermal growth factor receptor (EGFR) agents (eg, cetuximab/panitumumab), molecularly targeted agents against *BRAF*, and immune checkpoint inhibitors, which may only be available in maximal settings. ASCO is planning a systematic review–based guideline on targeted systemic therapy (non–resource stratified). Investigators are studying additional types of systemic therapy in maximal settings (see Future Directions section). Recently approved chemotherapeutic agents trifluridine/tipiracil may be available in some maximal settings. Systemic therapies are described in the following sections, as first-line, second-line, and third-line and beyond treatment options.

### CLINICAL QUESTION 2

What are the optimal **first-line systemic treatments** for patients with late-stage colorectal cancer?

The primary recommendations for treatment options in first line for metastatic colorectal cancer are found in Table 6; they are the same for curative intent and palliative intent and depend on resources available.

### Recommendation 2.13

For patients treated with oxaliplatin-based doublet or triplet chemotherapy ± anti-VEGF therapy (bevacizumab), it is reasonable to discontinue oxaliplatin after a period of induction (approximately 4 months; ie, 8 cycles) if the disease is stable or responding and continue maintenance single-agent fluoropyrimidine ± anti-VEGF therapy (bevacizumab) until symptomatic or radiologic progression. At the time of progression, reintroduction of the first-line therapy or a second-line therapy may be discussed (Strength: moderate; Setting: maximal).

### Recommendation 2.14

Patients with metastatic colorectal cancer with metachronous metastases, who received prior oxaliplatin-based chemotherapy for early-stage disease (resectable) within ≤ 12 months of diagnosis of metastatic disease, should receive doublet irinotecan-based chemotherapy in situations where doublet chemotherapy is recommended. For patients with metastatic colorectal cancer with metachronous metastases who received oxaliplatin-based chemotherapy for early-stage disease more than 12 months prior to the diagnosis of metastatic disease, all recommendations listed in Table 6 apply (Strength: strong; Setting: enhanced/maximal).

#### Discussion.

The first set of treatment recommendations (Table 6) state that clinicians should recommend doublet chemotherapy with fluoropyrimidine (FU or capecitabine) and oxaliplatin (FOLFOX or CAPEOX) or FU and irinotecan (FOLFIRI) for patients able to tolerate intensive chemotherapy and when resources are available.^34,35^ Using doublet chemotherapy is supported by strong evidence, according to most guidelines.^10,12,18,20^ For patients unable to tolerate intensive chemotherapy, or in limited-resource settings where it should be available, FU/leucovorin or capecitabine are acceptable treatment options.^36^ Doublet chemotherapy is not available in basic-, and typically not available in limited-resource settings. Capecitabine may not be cost effective in resource-constrained settings.^37^

#### Basic-resource settings.

No systemic chemotherapy is typically available in basic-resource settings for patients with advanced colorectal cancer. Patients should be offered palliative care according to ASCO Palliative Care Guidelines^22,23^ and, when possible, referred for treatment in less resource-constrained medical settings. The goal of palliative care is to prevent patient suffering.

#### Limited-resource settings.

For patients with late-stage CRC, single-agent chemotherapy with FU/leucovorin or with capecitabine should be available in limited-resource settings. Treatment with doublet chemotherapy (Table 6) should be offered when available. The use of FU/leucovorin or capecitabine is supported by NCCN, Cancer Council Australia, and ESMO guidelines.

#### Maximal- and enhanced-resource settings.

Doublet chemotherapy (FOLFOX or CAPEOX or FOLFIRI) is recommended due to increased efficacy compared with single-agent chemotherapy.^10,12,18,38,39^ In regions with fewer resource constraints, such as enhanced and maximal settings, doublet or triplet (FOLFOXIRI) chemotherapies should be available. NCCN, ESMO, and Cancer Council Australia guidelines advise that triplet chemotherapy (FOLFOXIRI) may be discussed for select patients, especially those appropriate for intensive chemotherapy, with large amount of disease burden, and when significant tumor shrinkage is the goal.^40,41^ For patients treated with oxaliplatin-based chemotherapy whose disease is stable/responding, it is reasonable to discontinue oxaliplatin after a period of induction therapy (approximately 4 months; ie, 8 cycles), and continue maintenance single-agent fluoropyrimidine until the patient becomes symptomatic or radiologic progression occurs. At that time, reintroduction of the first-line therapy or a second-line therapy can be discussed.^10,12,18^

Targeted therapies such as anti-VEGF and anti-EGFR agents may be added to doublet chemotherapies in maximal settings. The recommendation to add the anti-VEGF antibody bevacizumab to chemotherapy is moderate, based on available guidelines and panel consensus. Anti-VEGF therapy may be added to the doublet or triplet chemotherapy, irrespective of molecular status of the cancer. While the evidence is strong and it is listed as an option by NCCN and ESMO, the absolute clinical benefit in addition to chemotherapy is modest.^42-45^ NICE does not recommend anti-VEGF therapy as cost effective for treatment of patients with late-stage CRC.^20^ See the Cost Implications section regarding cost effectiveness of treatments in selected resource-constrained countries.

If molecular testing results for *RAS* (*KRAS*/*NRAS*) are available, this guideline provides recommendations according to the status of these markers. In maximal settings, for patients with left-sided colon cancer and known *KRAS*/*NRAS* wild type (WT) molecular status, anti-EGFR antibodies such as cetuximab or panitumumab may be added to chemotherapy doublet, with a moderate-strength recommendation. However, patients with right-sided colon cancer and *RAS* WT status should not be offered treatment with anti-EGFR antibodies in the first-line setting. Anti-EGFR therapies have increased response rates and conversion from unresectable to resectable metastatic disease when added to chemotherapy with FOLFOX or FOLFIRI for patients with *RAS* wild type,^46,47^ but more recent data suggest that benefit with anti-EGFR therapies seems to be limited to patients whose primary tumors are left sided.^10,12,18,20^ This is discussed in the cost-effectiveness literature^48-50^; however, some of this literature indicates limited cost effectiveness. Cancer Council Australia guidelines consider cautious use of anti-EGFR in addition to chemotherapy.

### CLINICAL QUESTION 3

What are the optimal **second-line systemic treatments** for patients with late-stage colorectal cancer who have received one prior line of therapy?

Recommendations for second-line treatment are in Table 7.

### CLINICAL QUESTION 4

What are the optimal **third-line systemic treatments** for patients with late-stage colorectal cancer who have received two prior lines of therapy? Recommendations for third-line and fourth-line metastatic colorectal cancer treatment are in Table 8.

Second- or third-line systemic therapies are only relevant to enhanced- and maximal-resource settings.

### Discussion

#### Basic- and limited-resource settings.

When possible, patients with good performance status, and after clinicians explain toxicities and risks versus benefits, may be referred to centers where enhanced or maximal resources are available, and patients may be offered second-line systemic therapies. Best supportive care should be provided to all patients according to ASCO Palliative Care Guidelines^22,23^ and others.

#### Enhanced- and maximal-resource settings.

Treatment of patients who have previously received one line of systemic therapy depends on what the patients received in first line (eg, if a patient has received oxaliplatin in first line, then irinotecan is recommended, based on NCCN, ESMO, Cancer Council Australia guidelines, and vice versa). The recommendations also take into consideration whether patients did or did not receive doublet/triplet chemotherapy, anti-VEGF agents, or anti-EGFR targeted therapies in the first-line setting. In maximal settings only, there are second-line recommendations based on molecular biomarkers (see Recommendations 2.4 and 2.5). As of this writing, Cancer Council Australia and ESMO have not reviewed the emerging immunotherapy literature, or the clinical trials with *BRAF* and/or MEK inhibitors for patients with *BRAF* V600E ([v-raf murine sarcoma viral oncogene homolog B1] valine at amino acid 600) mutations; this Expert Panel is aware of this (see Future Directions). Among the included source guidelines published in the included date parameter, none yet recommended immune checkpoint inhibitors or therapies targeting *BRAF*/MEK pathways with or without anti-EGFR agents, depending on histologic and molecular profiles. The Expert Panel is aware of the NCCN 2019 guideline version discussion of these agents.

The safety and efficacy of selective combination therapy for *BRAF* V600E-mutant metastatic colorectal cancer is being evaluated in an open-label, randomized, three-arm, phase III trial^51^ (BEACON Colorectal cancer trial; ClinicalTrials.gov identifier: NCT02928224; European Union Clinical Trials Register identifier: EudraCT2015-005805-35). Given that the literature search for this guideline excluded abstracts and news releases, it is outside the scope to fully review these data, which may only apply to certain maximal-resource settings. A future ASCO systematic review–based guideline on targeted therapy for patients with mCRC is planned and may review data from this trial.

Clinical trials are underway in the use of immunotherapy in maximal-resource settings, and the aforementioned systematic review–based ASCO guideline may review the primary literature for targeted therapies in maximal settings, which is outside of the scope of this guideline.

### CLINICAL QUESTION 5

What are selected **liver-directed therapy options** for patients with late-stage colorectal cancer and liver metastases? Recommendations regarding the treatment of patients with liver metastases are in Table 9.

**TABLE 9:**
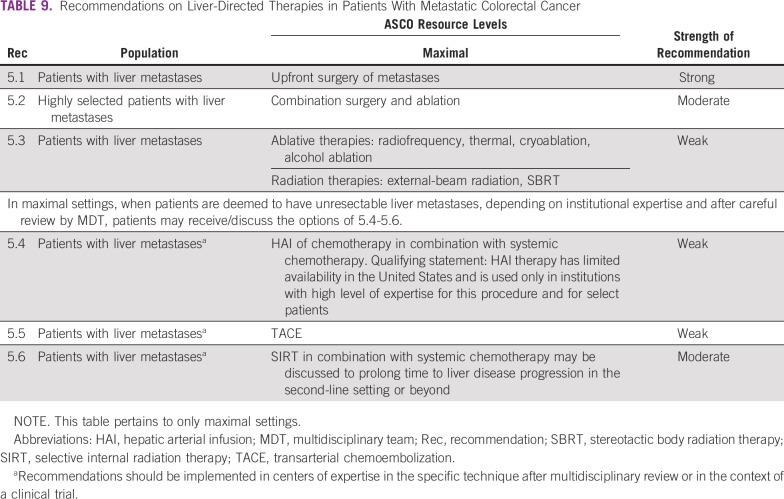
Recommendations on Liver-Directed Therapies in Patients With Metastatic Colorectal Cancer

### Discussion

Due to the lack of imaging in resource-constrained settings as well as the presumption of lack of these treatments in the other settings, this section is only relevant to maximal-level resource settings. The liver and lung are the most common sites of metastasis, and a small fraction (approximately 10% of patients with liver metastases) have potentially resectable disease, especially when the metastases develop metachronously.^52^ An MDT can determine the resectability of liver or lung metastases, including surgical consultation with experienced hepatic and/or cardiothoracic surgeons who determine the likelihood of achieving complete resection with negative margins and maintaining adequate liver reserve^10,21^ (30% future liver remnant required, or remnant to liver body weight ratio > 0.5,^12^ for liver resections) and complete resection of lung metastases based on the anatomic location and the extent of disease with maintenance of adequate function. In either case, the primary tumor must be resectable for curative intent (R0 resection margins). Several studies indicate that select patients who undergo surgery for liver metastases have prolonged survival (median 5-year survival, 38%), and some patients may be cured.^53,54^ When metastases from CRC also occur in the lung or other extrahepatic sites, combined or staged (sequential) surgeries of all sites of disease is a treatment element for some select patients, with favorable long-term outcomes.^55-58^ The Expert Panel elected to make recommendations specific to liver metastases.

Recommendations should be implemented in centers of expertise in the specific technique after multidisciplinary review or in the context of a clinical trial. In maximal settings only, when patients are deemed to have unresectable liver metastases, depending on institutional expertise and after careful review by an MDT, patients and clinicians may discuss the options in Recommendations 5.4-5.6. Hepatic arterial infusion therapy has limited availability in the United States and is used only in institutions with a high level of expertise for this procedure and for select patients.

#### Issues specific to patients with metastatic rectal cancer and primary-site radiation therapy.

In enhanced and maximal settings only, in patients with metastatic rectal cancer, the role of radiation therapy to the primary site is palliative unless the patient has oligometastatic disease with complete response to chemotherapy. Palliation is typically required for pain, bleeding, and/or obstruction. If the primary site disease is symptomatic (for example, with obstruction or bleeding), surgery, radiation, and/or chemotherapy should be discussed in the MDT while keeping the patient’s goals of care in mind. Although there is no high-quality literature on this topic, the MDT would determine the dose of radiation by the overall prognosis of the patient and burden of metastatic disease. Notably, radiation infrastructure is not routinely available in basic and limited settings. In these settings, the primary options are maximizing systemic therapy and medical pain management; when feasible, clinicians may also refer patients to higher-level facilities for radiation.

There is limited prospective evidence on the treatment of patients with unresectable rectal cancer with symptomatic primaries. This guideline bases these specific recommendations on the Cancer Council Australia, ESMO, and NCCN 2018 guidelines (Table 7).

### CLINICAL QUESTION 6

What is a summary of the optimal treatments for patients with late-stage colorectal cancer?

A summary of treatment options based on resource settings is described in Table 10. The primary evidence for Table 10 was from the Cancer Council Australia, ESMO, and NCCN guidelines.

**TABLE 10:**
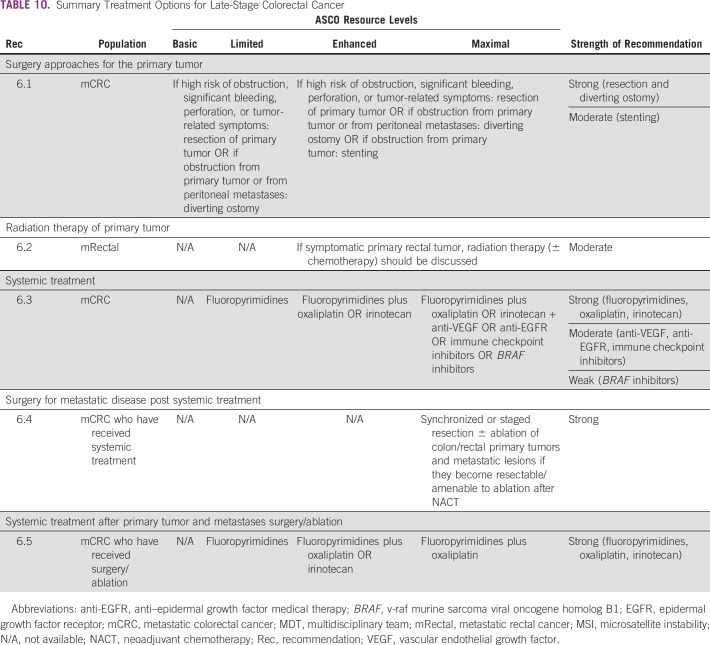
Summary Treatment Options for Late-Stage Colorectal Cancer

### CLINICAL QUESTION 7

What is the optimal on-treatment monitoring/surveillance and post-treatment follow-up strategy for patients treated for mCRC?

Recommendations are in Table 11.

**TABLE 11:**
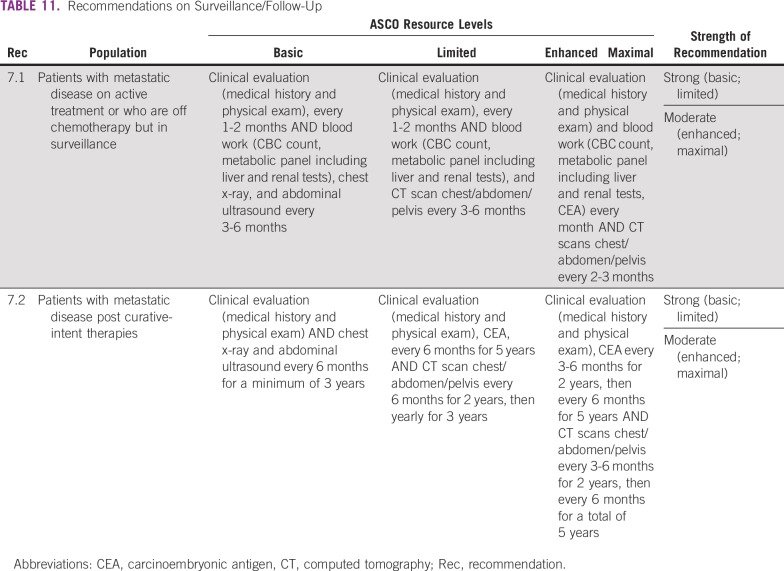
Recommendations on Surveillance/Follow-Up

### Discussion

The primary aim for surveillance is to prevent toxicity and to promote long-term survival with improved quality of life through early detection of progressive, local, or distant recurrent disease while minimizing false positives. It is critical to know all sites of disease that are being treated and to show documentation of clinical benefit, and toxicities, prior to each intervention.

Since some unresectable metastatic colorectal cancers can be relatively indolent, it is also very important to monitor patients on therapy for evidence of progression or toxicity on a regular basis to avoid overtreatment and the use of scarce resources with no evidence of benefit.

Five-year disease-free survival rates after aggressive surgical resection of metastatic disease in the lung, liver, or pelvis are roughly 40%, which also means that one in three patients will have a recurrence and ultimately die of metastatic disease.^59^

In all settings, the intensity of surveillance for recurrent or progressive disease should be based on the options available for the patient, should recurrence or progression be found. The evidence on the optimal frequency and/or intensity is limited. If local or metastatic surgery is an option, either due to the patient’s setting or if they have the resources to go to a maximal-resource setting, then maximal surveillance recommendations should be applied. The general condition and performance status of the patient must always be assessed before and during any treatment as well as before any “surveillance” imaging is offered. If the patient is unlikely to benefit from treatment due to poor performance status, then supportive care and palliative care are most appropriate in any setting. Chemotherapy in the last days of life is not associated with a survival benefit, and it may cause harm by decreasing quality of life and increasing costs. As a result, death within 30 days of chemotherapy has been used as a quality indicator for cancer care.^60-62^ Both ESMO and ASCO have published position statements encouraging discussions about the appropriate cessation of chemotherapy; however, implementation of such recommendations has been limited.^63^

Patient surveillance after curative resection will vary, and there is no validated consensus outside of maximal-resource settings, where the standard is to image the sites of resected disease every 3-6 months for 2 years, then every 6 months for a total of the first 5 years after diagnosis. Surveillance for recurrence of presenting symptoms can be done in any resource setting, but optimal frequency is not established. Once recurrence has been identified, management can be complex, and clinicians’ treatment decisions may benefit from specialized multidisciplinary input via cell phone and internet video conference calls, as done in Project ECHO or similar telementoring platforms.^64,65^

#### Basic-resource settings.

In a basic-resource setting, if a patient has completed curative resection of oligometastatic disease in the liver or lung, physical exam, review of symptoms, and basic imaging are appropriate, and referrals may be discussed or proposed depending on the scenario at the time that suspicion for recurrence arises.

#### Limited-resource settings.

If patients are being treated for unresectable metastatic disease, it is important to evaluate the patient clinically at least every 2 months with physical exam and thorough review of systems and evaluation of performance status. It should be very evident where disease is and which symptoms are being palliated with documentation of achieved palliation. Blood work should be monitored for signs of worsening anemia, liver function, or kidney function.

The combination of carcinoembryonic antigen (CEA) and CT scan has not shown additional value in reducing recurrence or mortality when compared with either one alone; therefore, in a limited setting, CT alone would also be a reasonable approach. For monitoring for disease progression, imaging done at baseline to determine the extent of disease (CT or x-ray and ultrasound) should be repeated at least every 2-4 months to monitor for response to therapy.

After curative resection of metastatic disease, suggested follow-up is clinical evaluation (medical history and physical examination) and CEA, every 6 months for 5 years, and imaging every 6 months for 2 years, then yearly for 5 years. Every 6 months the clinician should make a thorough assessment of the patient’s condition, comorbidities, and resources for treatment should they find recurrence before any imaging is done.

#### Maximal- and enhanced-resource settings.

In enhanced and maximal settings, access to potentially curative interventions in the setting of recurrent, but still resectable, metastatic disease is possible. Therefore, imaging should be done every 3-6 months for the first 2 years, then annually for up to 5 years, as most recurrences occur in the first 5 years. PET scans for surveillance are not routinely recommended in some guidelines due to risks of false positives but do appear in the Cancer Council Australia and NCCN guidelines. In general, the PET scan is most sensitive and specific when used to follow up on abnormalities seen on CT or ultrasound. PET/CT can be discussed in those patients whose cancer is potentially surgically resectable or amenable to potentially curable local directed therapy, after careful review by an MDT. A colonoscopy should also be performed for any clinical signs/symptoms of resectable recurrence.^66^

End-of-life cancer care is a significant challenge in any setting, and many doctors report a feeling of failure when transitioning to exclusively palliative treatment.^63^ Especially in basic and limited settings, the best timing and use of surgical and medical interventions is unclear. Additional training on difficult conversations and explaining risks as compared with benefits is essential to maximize the use of limited resources for those who benefit the most.

## SPECIAL COMMENTARY

### Multimodality Treatment

First-line treatment options for patients with late-stage colorectal cancer are the same whether used for curative intent or palliative intent and depend on resources available. Curative-intent treatment is determined by review by an MDT including surgical oncology experts and requires evaluation of all diagnostic and staging tests available. For curative-intent treatments, the order of treatment interventions may be: (1) surgery first followed by adjuvant therapy, or (2) NACT followed by surgery, followed or not by adjuvant therapy (neoadjuvant and adjuvant therapy = perioperative therapy). Moderate evidence suggests a duration of NACT prior to surgery of 2-3 months to prevent hepatotoxicity and a total duration of perioperative therapy of 6 months.^10,12,26,67-69^ For patients who did not receive NACT, moderate evidence recommends 6 months of adjuvant chemotherapy. The sequence of treatments is determined by the MDT after ascertaining resectability and resources available. When clinicians in resource-constrained settings believe that a patient may benefit from medical and surgical treatments with curative intent, due to limited amount(s) of metastatic disease visible on imaging and good overall performance status, they should refer the patient to a center with MDT and maximal resources where available.

Routine resection of primary colorectal tumors is not recommended for patients with metastases. MDTs may discuss resection of the primary tumor (colectomy) prior to starting systemic treatment of metastatic disease for patients with a symptomatic primary tumor (imminent risk of obstruction, significant bleeding, perforation, or other significant tumor-related symptoms) and synchronous metastatic disease.^10^ Patients who are not candidates for surgery may be offered other palliative treatment options, including a surgical bypass, diverting ostomy, radiation therapy, or stenting.

As few as 10% of patients with limited metastatic disease have potentially resectable disease at diagnosis. These patients may proceed directly to surgery with curative intent if deemed resectable for both the primary and metastatic tumor sites, after review by the MDT. Surgery may occur synchronously for the primary and metastatic sites or as a staged/sequential approach. Surgery for metastases is the preferred approach, but, when not feasible, local therapies such as ablation or radiation therapy may be discussed (Table 5). For patients with rectal primary tumors, prior to surgical resection, chemoradiotherapy or short-course radiation to the primary tumor should be discussed to reduce the chance of local recurrence (Table 6). After surgery, patients may be offered adjuvant chemotherapy, typically for a 6-month course, with a moderate level of evidence. ESMO did not find strong evidence for adjuvant chemotherapy. Some studies show improved progression-free survival but not OS.^69-71^ Another option for patients with potentially resectable metastatic disease is NACT,^10,12,18^ usually for 2-3 months, followed by synchronous or staged surgery of the primary tumor and metastatic disease, followed by adjuvant chemotherapy. The NCCN 2018 guidelines recommend 6 months of total perioperative chemotherapy (perioperative therapy consists of both neoadjuvant and adjuvant therapy).

As many as 90% of patients with mCRC have unresectable disease. Less than 20% of patients with late-stage disease may have disease that may become resectable after receiving initial treatment with systemic therapy. Systemic therapy options are the same for locally advanced unresectable and for mCRC (Tables 6, 7, 8, and 10). It is critical that MDTs review the cases of these patients to determine the best sequence of therapies and eligibility for curative-intent surgery. It is important to review the goals of care with the patient: curative intent versus palliative. If obtaining a response to treatment is important, such as for converting initially unresectable localized or metastatic disease to resectable disease, using the combination regimens available with highest response rates should be a strong consideration. If response to therapy is observed on follow-up imaging, surgical resection of the primary tumor and metastatic sites may be discussed either as synchronous operations or as a sequential approach, after review by the MDT.

If surgical resection of all metastatic sites is not possible, a combination of resection and other local-directed therapies such as those listed in Table 9 (liver-directed therapies in patients with metastatic colorectal cancer) may be discussed (in maximal settings). All curative-intent surgeries and radiotherapy are likely only available in maximal settings, with presumption of lack of these treatments in the other settings. Patients with metastatic rectal cancer may benefit from chemoradiation or short-course radiation of the primary tumor with palliative intent, in cases where local disease control is necessary to alleviate pain, bleeding, or obstruction.

## PATIENT AND CLINICIAN COMMUNICATION

For recommendations and strategies to optimize patient-clinician communication, see Patient-Clinician Communication: American Society of Clinical Oncology Consensus Guideline.^72^ Communication should be culturally specific and informed by cultural competence; it is not possible for this guideline to address all situations.

## COST IMPLICATIONS

Discussion of cost can be an important part of shared decision making.^73^ Clinicians should discuss the use of less-expensive alternatives with patients when it is practical and feasible for treatment of the patient’s disease and there are two or more treatment options that are comparable in terms of benefits and harms.^73^

Patient out-of-pocket costs may vary depending on resource setting. When discussing financial issues and concerns, patients should be made aware of any financial counseling services available to address this complex and heterogeneous landscape.^73^

As part of the guideline development process, ASCO may opt to search the literature for published cost-effectiveness analyses that might inform the relative value of available treatment options. Excluded from consideration are cost-effective analyses that lack contemporary cost data and/or are industry sponsored. There were five cost-effectiveness analyses in non-maximal settings identified by conducting a search of PubMed and the Tufts Cost-Effectiveness Analysis Registry.

Published cost-effectiveness analyses of molecularly targeted agents, primarily in combination, found the addition of these agents to older regimens (eg, including capecitabine and oxaliplatin-containing regimens) was not cost effective in LMICs, including in literature found from Brazil, China, and Iran. Three studies of combinations of cetuximab found they were not cost effective.^48-50^ One of the studies found the same for panitumumab.^50^ In an example regarding VEGF targeted therapy, Ungari et al^74^ conducted a cost-effectiveness analysis of adding bevacizumab to capecitabine and oxaliplatin (XELOX) in Brazil and found it was not cost effective. The only study found with a cost-effective regimen was a modified first-line FU, leucovorin, and oxaliplatin (FLOX) regimen with lower-dose leucovorin.^75^ Older regimens available in generic form may be cost effective (source: Dr. Ali Shamseddine, personal communication, September 2018; Data Supplement).

## OPEN COMMENT

The draft recommendations were released to the public for open comment from July 17 through July 31, 2019. Response categories of “Agree as written,” “Agree with suggested modifications,” and “Disagree. See comments” were captured for every proposed recommendation with 10 written comments received. A total of 88% of the 10 respondents’ responses either agreed or agreed with slight modifications to the recommendations, and 12% of the respondents’ responses disagreed. Expert Panel members reviewed comments from all sources and determined whether to maintain original draft recommendations, revise with minor language changes, or consider major recommendation revisions; original draft recommendations were maintained. In general, any changes to draft ASCO guidelines based on Open Comment are incorporated prior to final Clinical Practice Guidelines Committee review and approval.

## GUIDELINE IMPLEMENTATION

ASCO guidelines are developed for implementation across health settings. Barriers to implementation include the need to increase awareness of the guideline recommendations among front-line practitioners and survivors of cancer and caregivers and also to provide adequate services in the face of limited resources. The guideline Bottom Line Box was designed to facilitate implementation of recommendations. This guideline will be distributed widely through the ASCO Practice Guideline Implementation Network. ASCO resource-stratified guidelines are posted on the ASCO Web site and most often published in the *Journal of Global Oncology* and a summary in the *Journal of Oncology Practice*.

## LIMITATIONS OF THE RESEARCH AND FUTURE RESEARCH

There were limitations on the evidence to inform some of the recommendations. There were limited guidelines systematically reviewing and/or finding high-quality published data on:

- Best treatment of patients with metastatic rectal cancer in resource-constrained settings
- Role of primary-site radiation therapy
- Liver metastases–directed therapies
- Lung metastases–directed therapies
- Use of imaging in liver metastases
- Role of targeted therapy, including patients who received anti-VEGF or anti-EGFR in first line
- Systematic review–based guidelines including *BRAF* targeted therapy and/or MEK targeted therapy
- Systematic review–based guidelines including immune checkpoint inhibitors (eg, nivolumab, pembrolizumab, ipilimumab).

### Future Directions

The authors were aware of the BEACON trial (the safety phase of a phase III trial). None of the vetted source guidelines reviewed and fitting this guideline’s date parameters included a trial on a *BRAF* V600E-targeted combination therapy intervention. This ongoing trial is an open-label phase III randomized clinical trial (RCT).^51^ At this writing, the efficacy phase of the RCT was ongoing. A future ASCO systematic review–based guideline may review published data from this trial. At that time, the Expert Panel will determine if this guideline should be updated to include those data.

Based on the Limitations and Future Directions, the Expert Panel suggests research and high-quality guideline development be conducted on these topics, especially with studies conducted in resource-constrained settings.

**ASCO believes that cancer clinical trials are vital to inform medical decisions and improve cancer care, and that all patients should have the opportunity to participate.**

## ADDITIONAL RESOURCES

More information, including a data supplement with additional evidence tables, slide sets, and clinical tools and resources, is available at www.asco.org/resource-stratified-guidelines. Patient information is available at www.cancer.net.

**Related ASCO Guidelines**

- Palliative Care in the Global Setting Resource-Stratified Guideline^23^ (http://ascopubs.org/doi/10.1200/JGO.18.00026)
- Patient-Clinician Communication^72^ (http://ascopubs.org/doi/10.1200/JCO.2017.75.2311)
- Early Detection for Colorectal Cancer Resource-Stratified Guideline^8^ (http://ascopubs.org/doi/10.1200/JGO.18.00213)
- Treatment of Patients with Early-Stage Colorectal Cancer Resource-Stratified Guideline^9^ (http://ascopubs.org/doi/10.1200/JGO.18.00214)

## Figures and Tables

**TABLE A1:**
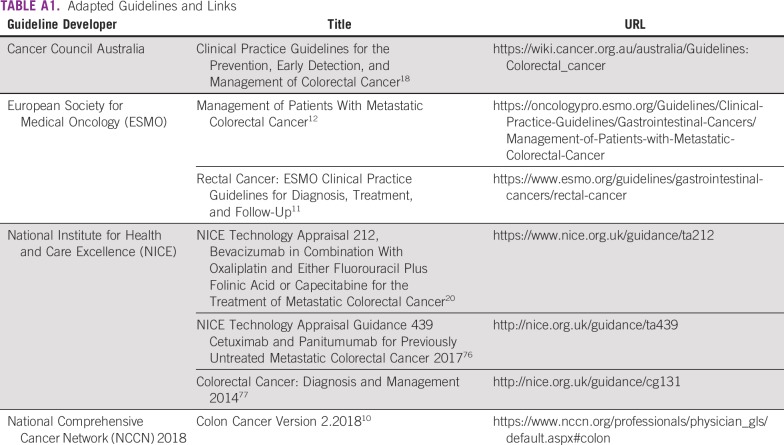
Adapted Guidelines and Links

**TABLE A2:**
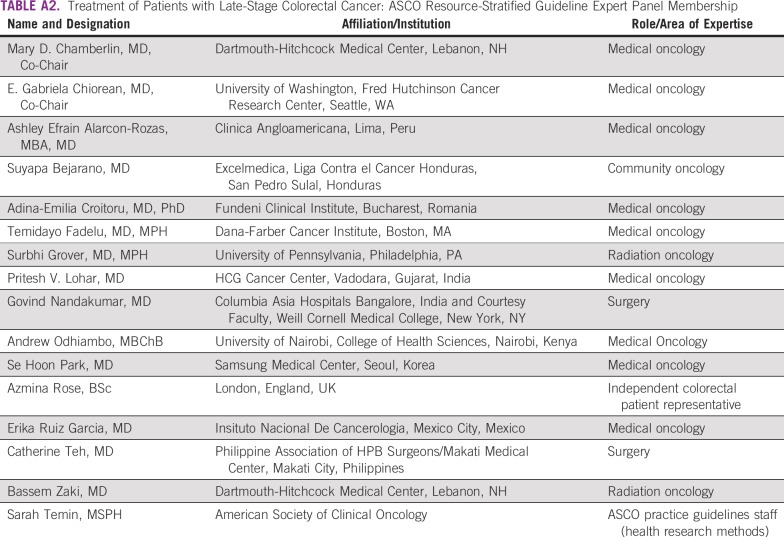
Treatment of Patients with Late-Stage Colorectal Cancer: ASCO Resource-Stratified Guideline Expert Panel Membership
